# Androgen Regulation of the Mesocorticolimbic System and Executive Function

**DOI:** 10.3389/fendo.2018.00279

**Published:** 2018-06-05

**Authors:** Daniel J. Tobiansky, Kathryn G. Wallin-Miller, Stan B. Floresco, Ruth I. Wood, Kiran K. Soma

**Affiliations:** ^1^Department of Psychology, University of British Columbia, Vancouver, BC, Canada; ^2^Djavad Mowafaghian Centre for Brain Health, University of British Columbia, Vancouver, BC, Canada; ^3^Neuroscience Graduate Program, University of Southern California, Los Angeles, CA, United States; ^4^Department of Integrative Anatomical Sciences, Keck School of Medicine of the University of Southern California, Los Angeles, CA, United States; ^5^Department of Zoology, University of British Columbia, Vancouver, BC, Canada

**Keywords:** 3β-hydroxysteroid dehydrogenase, aromatase, cognition, Cyp17a1, estradiol, neurosteroid, dehydroepiandrosterone, LC–MS/MS

## Abstract

Multiple lines of evidence indicate that androgens, such as testosterone, modulate the mesocorticolimbic system and executive function. This review integrates neuroanatomical, molecular biological, neurochemical, and behavioral studies to highlight how endogenous and exogenous androgens alter behaviors, such as behavioral flexibility, decision making, and risk taking. First, we briefly review the neuroanatomy of the mesocorticolimbic system, which mediates executive function, with a focus on the ventral tegmental area (VTA), nucleus accumbens (NAc), medial prefrontal cortex (mPFC), and orbitofrontal cortex (OFC). Second, we present evidence that androgen receptors (AR) and other steroid receptors are expressed in the mesocorticolimbic system. Using sensitive immunohistochemistry and quantitative polymerase chain reaction (qPCR) techniques, ARs are detected in the VTA, NAc, mPFC, and OFC. Third, we describe recent evidence for local androgens (“neuroandrogens”) in the mesocorticolimbic system. Steroidogenic enzymes are expressed in mesocorticolimbic regions. Furthermore, following long-term gonadectomy, testosterone is nondetectable in the blood but detectable in the mesocorticolimbic system, using liquid chromatography tandem mass spectrometry. However, the physiological relevance of neuroandrogens remains unknown. Fourth, we review how anabolic-androgenic steroids (AAS) influence the mesocorticolimbic system. Fifth, we describe how androgens modulate the neurochemistry and structure of the mesocorticolimbic system, particularly with regard to dopaminergic signaling. Finally, we discuss evidence that androgens influence executive functions, including the effects of androgen deprivation therapy and AAS. Taken together, the evidence indicates that androgens are critical modulators of executive function. Similar to dopamine signaling, there might be optimal levels of androgen signaling within the mesocorticolimbic system for executive functioning. Future studies should examine the regulation and functions of neurosteroids in the mesocorticolimbic system, as well as the potential deleterious and enduring effects of AAS use.

## Introduction

**Figure 1:**
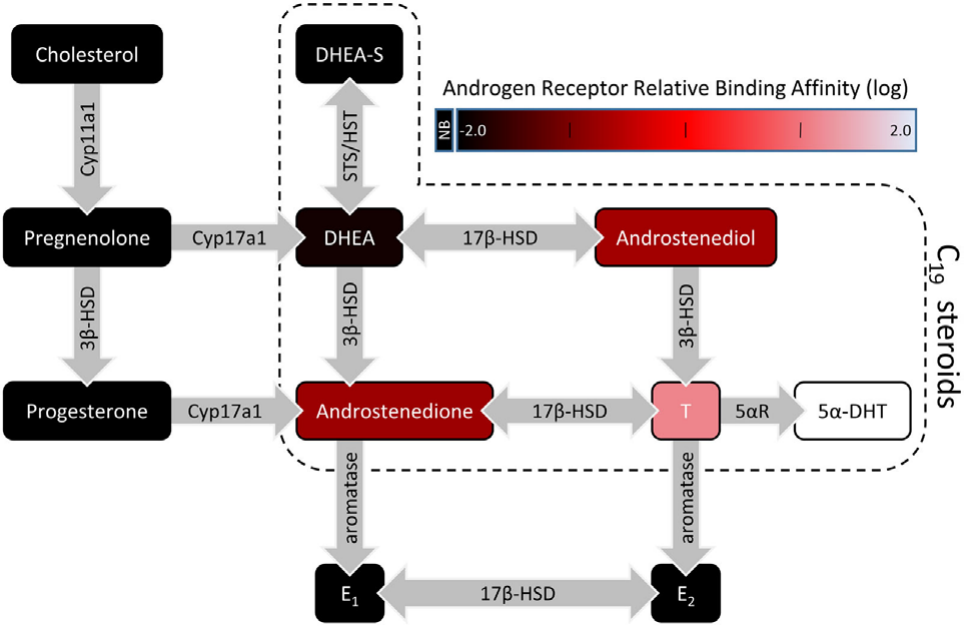
A simplified illustration of the steroidogenic pathway with a focus on C_19_ steroids. For C_19_ steroids, fill color represents the relative binding affinity to the androgen receptor (4–6). Steroidogenic enzymes are represented by the gray arrows. Abbreviations: 3β-HSD, 3β-hydroxysteroid dehydrogenase/isomerase; 5αR, 5α-reductase; 5α-DHT, 5α-dihydrotestosterone; 17β-HSD, 17β-hydroxysteroid dehydrogenase; DHEA, dehydroepiandrosterone; DHEA-S, dehydroepiandrosterone sulfate; E_1_, estrone; E_2_, 17β-estradiol; HST, hydroxysteroid sulfotransferase; STS, steroid sulfatase; NB, no binding.

Berthold first reported the masculinizing effects of a bloodborne “substance” produced by the testes in male chicks (1). This substance is now known to belong to a class of steroids called androgens, which are synthesized by the male gonads and released into the circulatory system to regulate development, physiology, and behavior. Endogenous androgens are 19-carbon (C_19_) steroids and include testosterone (T) and its metabolite 5α-dihydrotestosterone (DHT), which have the most pronounced androgenic effects. Other C_19_ steroids include T precursors such as dehydroepiandrosterone (DHEA) and androstenedione (Figure 1). Androgen synthesis from cholesterol occurs in the Leydig cells of the testes, stromal, and thecal cells of the ovaries, and the zona reticularis of the adrenal cortices in some mammalian species (2, 3).

Numerous studies examining the effects of gonadectomy (GDX), androgen receptor (AR) antagonists, androgen synthesis inhibitors, androgen replacement, and administration of supraphysiological amounts of androgens [i.e., anabolic-androgenic steroids (AAS)] demonstrate that androgens are critical for reproductive behavior [reviewed in Ref. (7)] and aggressive behavior [reviewed in Ref. (8, 9)]. However, recent research has revealed that more complex behaviors and cognitive processes, such as executive function, are also regulated by androgens. We will review research that examines the role of androgens in regulating the neural circuitry that mediates executive function and behaviors associated with executive function.

In the first section “The mesocorticolimbic system and executive function,” we give a brief overview of the mesocorticolimbic system and its involvement in various executive functions. In the second section, we describe evidence for the presence of sex steroid receptors in the mesocorticolimbic system. In the third section, we summarize recent work that provides strong evidence for local synthesis of androgens and estrogens within the mesocorticolimbic system. In the fourth section, we discuss how AAS modulate the mesocorticolimbic system. In the fifth section, we explore how androgens alter neurochemical signaling and cytoarchitecture in nodes of the mesocorticolimbic system. Finally, in the last section, we review preclinical and clinical studies demonstrating that GDX, AAS, and perhaps local production of androgens influence executive functions such as behavioral flexibility and inhibitory control. Like many other neuromodulator systems, androgen signaling levels are likely maintained at particular levels within different brain regions to achieve optimal executive function.

## The Mesocorticolimbic System and Executive Function

**Figure 2:**
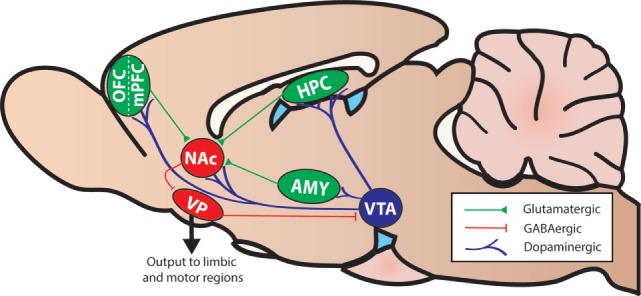
A simplified diagram of the mesocorticolimbic system and associated structures in the rodent brain (sagittal view). Abbreviations: AMY, amygdala; HPC, hippocampus; NAc, nucleus accumbens; mPFC, medial prefrontal cortex; OFC, orbitofrontal cortex; VP, ventral pallidum; VTA, ventral tegmental area.

Executive functions are a collection of cognitive operations that interact to facilitate selection and implementation of behaviors to attain chosen goals. More basic operations include selective attention, inhibitory control, and working memory (i.e., temporary maintenance and manipulation of information). These operations work in concert with those processed by other mnemonic, affective, and motivational systems to regulate more complex processes such as cognitive flexibility and cost/benefit decision making. It is well established from lesion and functional imaging studies in humans and non-human animals that various aspects of executive functioning are critically dependent on different regions of the prefrontal cortex (PFC) and its interactions with striatal regions, including the nucleus accumbens (NAc; Figure 2).

The PFC and the NAc receive dopamine (DA) input from the ventral tegmental area (VTA) in the midbrain, and DA transmission within these regions plays a key role in facilitating both basic and more complex functions mediated by these circuits. Thus, the seminal findings of Brozoski et al. (10) revealed that DA depletion in the frontal lobes of monkeys markedly impairs working memory, and subsequent psychopharmacological studies revealed that these functions are dependent primarily on PFC D_1_ receptor (D_1_R) activity [reviewed in Ref. (11)]. Different forms of cognitive flexibility are also dependent on DA activity within the frontal lobes and/or striatal regions. For example, shifts between strategies, rules, or attentional sets are dependent on DA transmission in both the medial prefrontal cortex (mPFC) and NAc. D_2_ receptors (D_2_R) in the PFC facilitate suppression of old strategies, whereas D_1_R in the PFC and NAc facilitate establishment and maintenance of new strategies (11–14). In comparison, reversal learning is a simpler form of cognitive flexibility, entailing a shift between stimulus–reinforcement associations (i.e., use the same basic strategy, but approach a different stimulus). The orbitofrontal cortex (OFC) plays a key role in mediating reversal learning in both primates and rats (15, 16). Reversal learning is generally unimpaired by global depletion of PFC DA (17), and DA input to dorsal striatal regions appears more crucial to this form of flexibility (18, 19).

Dopamine transmission in prefrontal–striatal circuitry also mediates evaluative functions entailing a choice between a smaller, readily available reward vs. a larger/more palatable reward associated with some form of cost, which can diminish the subjective value of objectively larger or more-preferred rewards. These forms of decision making are exquisitely sensitive to manipulation of DA transmission, in that systemic treatment with DA antagonists reduces preference for larger rewards associated with a greater effort cost or uncertainty (20–22). However, the mechanisms through which DA regulates choice behavior can vary across different nodes of the mesocorticolimbic circuit. For example, blockade of D_1_R, but not D_2_R, in the NAc reduces risky choice (23), whereas blockade of either receptor in the NAc diminishes preference for more preferred rewards associated with a greater effort cost (24). Likewise, blockade of D_1_R, but not D_2_R, in different subregions of the mPFC shifts preference away from more costly rewards (25, 26) and also makes animals more risk-averse (27). Yet, blockade of PFC D_2_R impairs modifications of decision biases in response to changes in risk/reward contingencies (27, 28). Collectively, these studies indicate that DA transmission within different nodes of the mesocorticolimbic system helps to refine different types of decision making by promoting choice toward larger, yet more costly, rewards, and modifying decision biases when cost/benefit contingencies change. The critical involvement of DA in various executive functions suggests that other signals that can influence DA signaling, such as sex steroids, may also influence these functions.

## The Mesocorticolimbic System Contains Sex-Steroid Receptors

Multiple lines of evidence indicate that receptors for sex steroids are present in the VTA, NAc, mPFC, and OFC. Here, we focus on the classical AR, the estrogen receptors (ER)α and ERβ, and more recently discovered membrane-associated androgen receptors (mAR) and ER (mER). We briefly discuss androgen metabolites that can act *via* allosteric binding sites on neurotransmitter receptors.

Androgens can act on target cells by binding to intracellular AR. Of the endogenous androgens, T and DHT have the highest binding affinities for AR, while DHEA, androstenedione, and androstenediol have weak binding affinities for AR [(4–6); Figure 1]. AAS have a wide range of binding affinities for AR, and users select different AAS according to the balance of desired anabolic (myotrophic) actions and unwanted side-effects (e.g., gynecomastia).

Androgens are lipophilic and non-polar, and thus they can pass through the blood–brain barrier and then the plasma membrane of cells to bind with AR in the cytosol. This ligand–receptor complex then dimerizes, is phosphorylated, and translocates to the cell nucleus, where the DNA-binding domain binds to a specific sequence of DNA called the hormone response element and acts as a transcription factor (29). Such genomic effects are responsible for many of the peripheral effects of androgens, such as enhancing muscle growth (30). ARs are also found in multiple brain regions. Generally, ARs are found in the highest concentrations in hypothalamic and limbic regions that regulate homeostatic functions, reproductive behaviors, and aggressive behaviors (31). For example, male mice with reduced AR in the nervous system show decreases in mating and aggression (32).

**Figure 3:**
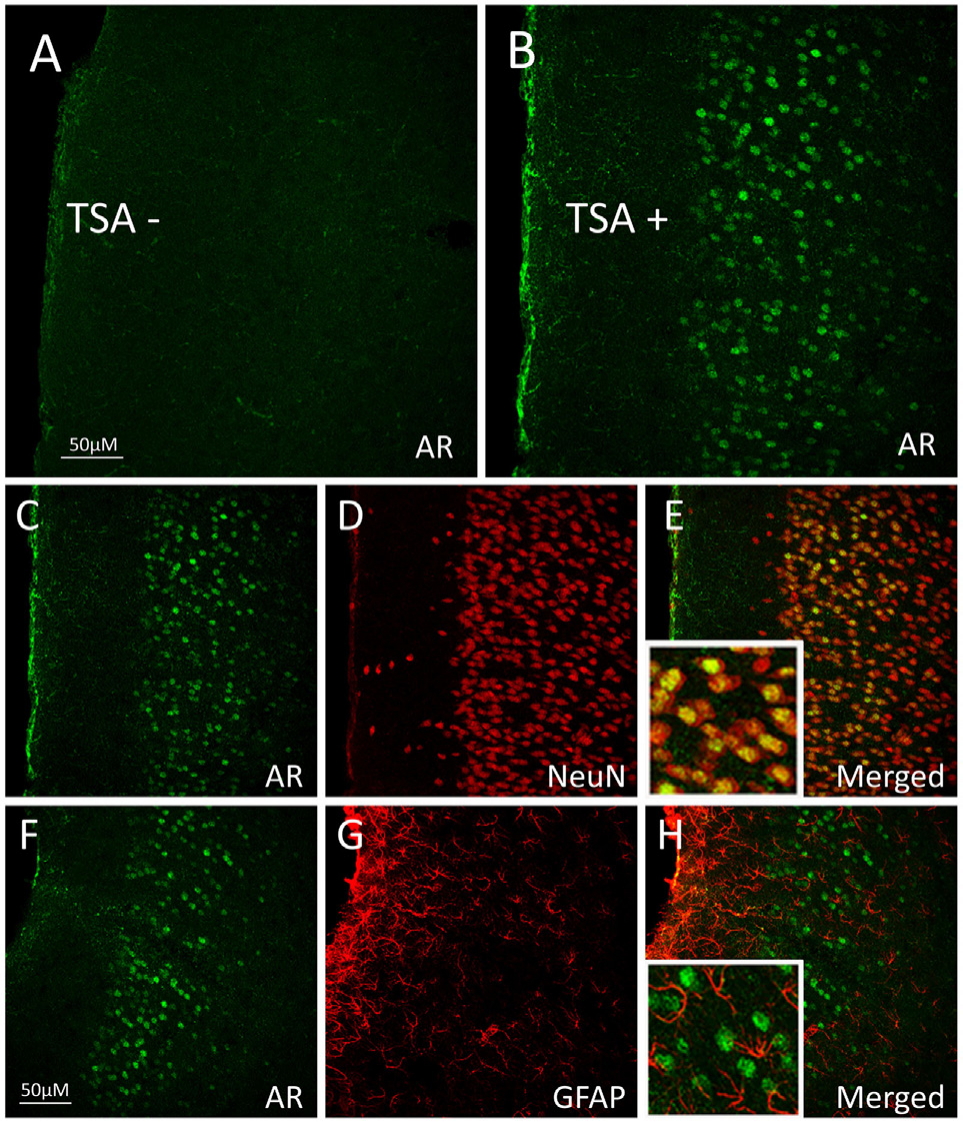
Androgen receptor (AR), neuronal nuclei (NeuN, neuronal marker), and glial fibrillary acidic protein (GFAP; glial marker) immunoreactivity (ir) in the medial prefrontal cortex (mPFC) of adult male rats. **(A,B)** Pseudocolored confocal photomicrographs of androgen receptor immunoreactivity (AR-ir) in coronal hemisections of the mPFC **(A)** without tyramide signal amplification (TSA−) and **(B)** with tyramide signal amplification (TSA+). TSA-enhanced detection of AR in the mPFC of male rats. **(C–E)** Confocal photomicrographs of mPFC with **(C)** AR-ir cells (green), **(D)** NeuN-ir cells (red), and **(E)** AR-ir and NeuN-ir cells merged. Cells that co-express AR-ir and NeuN-ir appear orange–yellow, suggesting that AR is primarily expressed in neurons. **(F–H)** Confocal photomicrographs of **(F)** AR-ir cells (green), **(G)** GFAP-ir cells (red), and **(H)** AR-ir and GFAP-ir cells merged. AR-ir is not co-expressed with GFAP. Adapted from Ref. (33); Reprinted by permission of SAGE Publications.

**Figure 4:**
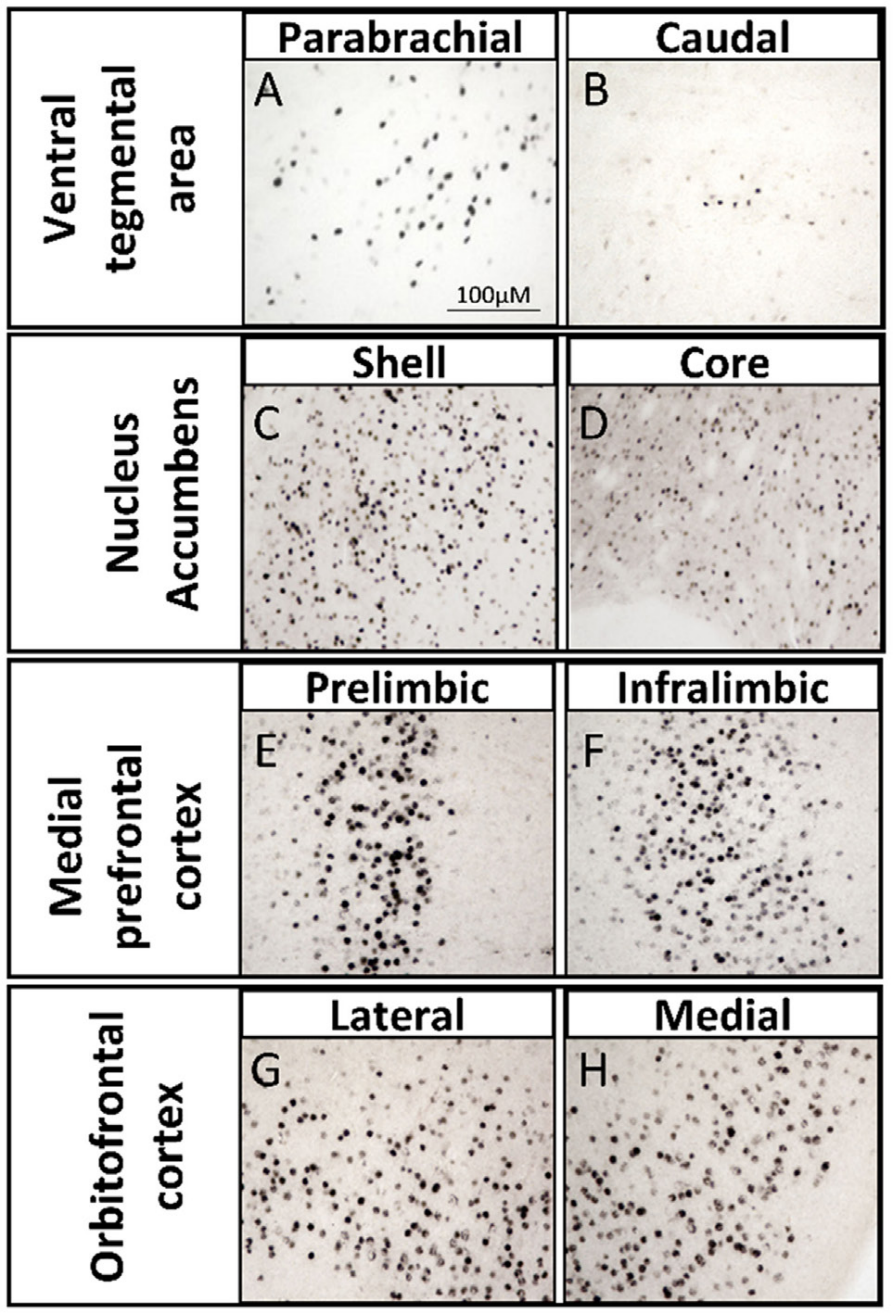
Brightfield photomicrographs depicting androgen receptor immunoreactivity (AR-ir) with tyramide signal amplification in nodes of the mesocorticolimbic system of adult male rats. AR-ir in the **(A)** parabrachial pigmented nucleus of the ventral tegmental area (VTA), **(B)** caudal VTA, **(C)** shell of the nucleus accumbens (NAc), **(D)** core of the NAc, **(E)** prelimbic subregion of the medial prefrontal cortex (mPFC), **(F)** infralimbic subregion of the mPFC, **(G)** lateral subregion of the orbitofrontal cortex (OFC), and **(H)** medial subregion of the OFC. Adapted from Ref. (45).

One way in which androgens might influence executive function is through direct actions on the mesocorticolimbic system. ARs are expressed in regions of the mesocorticolimbic system, albeit at lower levels than in the hypothalamus. In particular, the VTA, NAc, and mPFC express low to moderate levels of AR in male and female rodents (33–37), non-human primates (38, 39), and humans (40). Using microdissected tissue from mesocorticolimbic nodes, we recently demonstrated AR mRNA in the VTA, NAc, and mPFC using sensitive and specific probe-based quantitative polymerase chain reaction (qPCR) assays (36). The presence of AR protein immunoreactivity (AR-ir) in these regions has also been reported; however, the number of AR per cell is low, which results in immunohistochemical staining that is faint, challenging to quantify, and easy to overlook (41). One reason is that, in extrahypothalamic regions, androgen receptor immunoreactivity (AR-ir) is often located in neuronal processes and not concentrated in neuronal nuclei. Nonetheless, there are many processes and nuclei that express AR in the cerebral cortex, which has been verified by immunoelectron microscopy (35, 41). By adding a Tyramide Signal Amplification (TSA) step in the immunohistochemistry protocol, we recently showed that AR-ir cells are present in the VTA, NAc, mPFC, and OFC [(33); Figures 3 and 4]. Double-label immunofluorescence coupled with confocal microscopy demonstrates that AR-ir cells in the PFC are neurons (Figure 3). In the VTA, AR-ir cells express tyrosine hydroxylase (TH), a marker of DA-synthetic neurons (42). Furthermore, perikarya in the VTA that project to the NAc and mPFC express AR (43). Of the VTA neurons that project to the prelimbic mPFC (mPFC-PL), the proportion of DAergic (TH-positive) efferents containing AR is higher in male rats than female rats (~30 vs <5%), but the proportion of TH-negative efferents containing AR is similar between males and females (44). Thus, androgens can influence the male mPFC *via* actions on these DAergic projection neurons (42). Taken together, these data suggest that AR are well positioned to modulate executive function.

In addition, T can be locally aromatized to E_2_ and bind to ER in the mesocorticolimbic system (Figure 1). Many brain regions contain aromatase, the enzyme that catalyzes the conversion of androgens to estrogens (36). Aromatase expression is high in the hypothalamus (46, 47), and aromatase is also present in other regions including the mesocorticolimbic system (see below). The VTA, NAc, and mPFC contain some cells that express ERα or ERβ in female and male rats (48, 49). However, the VTA neurons that project to the NAc do not express ERβ. Instead, in both sexes, VTA neurons that express ERβ project principally to the ventral caudate putamen and amygdala (43). VTA neurons that project to mPFC-PL (TH-positive and TH-negative) lack ERα and less than 10% contain ERβ (44). In general, in female and male rodents, the NAc also has little intracellular ERα and ERβ (49–51).

In addition, androgens can modulate the mesocorticolimbic system through other mechanisms. First, hypothalamic nuclei that have high concentrations of AR, ERα, and ERβ directly innervate mesocorticolimbic nodes and influence DA release. For example, the medial preoptic area is rich in AR and ERs and projects to the VTA and modulates DAergic neurons (52–54). Second, mAR and mER might mediate the rapid, nongenomic effects of androgens in the mesocorticolimbic circuit. Two possible candidates for mAR are ZIP9 and GPRC6A (55–57). However, no studies have examined ZIP9 or GPRC6A in mesocorticolimbic nodes, and whole-brain analyses have not reported either transcript in the VTA, NAc, or mPFC in mice (58, 59) or humans (40). In addition, AR variants have been found in neuronal lipid rafts (60). Alternatively, the G protein–coupled estrogen receptor 1 (GPER1; formerly known as GPR30) is present in the VTA, NAc, and, to a lesser extent, the PFC in rats and humans (61–63). Thus, systemic and locally synthesized estrogens could act on the mesocorticolimbic system *via* GPER1. Third, some C_19_ steroids can rapidly (milliseconds to seconds) modulate neuronal excitability *via* allosteric binding sites on neurotransmitter receptors, voltage-gated channels, and neurotrophin receptors [reviewed in Ref. (64)]. For example, the γ-aminobutyric acid (GABA)-gated chloride channel GABA_A_ receptor (GABA_A_R) and the glutamate-gated sodium/calcium channel *N*-methyl-D-aspartate receptor are sensitive to allosteric regulation by DHEA, DHEA-S, and 3α-androstanediol (65, 66).

## The Mesocorticolimbic System Locally Synthesizes Androgens

Our understanding of the role of androgens in the brain changed dramatically with the first suggestion of steroid synthesis in the rodent brain. Baulieu, Robel, and colleagues (67, 68) originally suggested that levels of DHEA and pregnenolone and their sulfoconjugates were higher in grossly dissected regions of the male rat brain (i.e., divided into the “anterior” and “posterior” brain) than in the serum. Moreover, GDX and adrenalectomy did not eliminate these steroids in the brain. Later, Liere and colleagues described how these findings were actually artifacts resulting from sample preparation, including oxidation of cholesterol in brain tissue (69). Recent studies, however, have shown that androgens are present at higher levels in several brain regions than in the blood in male rats [e.g., (36)], are directly synthesized in the brain in female and male rats [e.g., (70, 71)], or metabolized in the brain [e.g., (72)]. Local production of neurosteroids serves to influence gene expression or neuron excitability in an intracrine, paracrine, autocrine, or synaptocrine manner under normal physiological conditions (73) or as a compensatory mechanism when circulating steroid levels are low (74).

The steroidogenic capacity of the brain is further corroborated by studies demonstrating that steroidogenic enzymes are present in the brain. In many of the initial studies, the lower sensitivity of Northern blots, *in situ* hybridization, immunohistochemistry, and even PCR was insufficient to detect some steroidogenic enzymes in the brain. For example, Goascogne and colleagues (75) attempted to detect Cyp17a1 (Figure 1), which catalyzes conversion of progestins into androgens, in the rodent brain *via* immunostaining, but it was not until 1995 that several groups detected *Cyp17a1* transcripts and protein in the brain (76–78), and even then only at very low levels or only in embryonic brains. Several labs did find other steroidogenic enzymes in the brain, including Cyp11a1 (76, 79) and aromatase (78). Guennoun and colleagues (80) detected mRNA and protein of 3β-hydroxysteroid dehydrogenase/isomerase (3β-HSD) in the hippocampus (HPC), hypothalamus, cerebellum, and cerebral cortex. Current techniques, particularly PCR, can detect all the enzymes necessary for androgen synthesis and metabolism in multiple regions in the male and female rat brain (81–84) and human brain (85, 86).

Little is known about the androgenic capacity of the mesocorticolimbic system, and even less about the physiological relevance of these locally produced steroids. Most studies have measured steroidogenic enzymes in gross neuroanatomical regions (e.g., cerebral cortex, HPC), without specific attention to mesocorticolimbic regions [e.g., (81, 87, 88)]. Specifically, Cyp11a1, Cyp17a1, and aromatase have been reported in the frontal cortex and midbrain or tegmentum of birds (89, 90), rodents (79), and humans [reviewed in Ref. (85)]. However, these reports have low spatial resolution, so steroidogenic enzyme levels specifically in the mPFC or VTA are unclear. Raab and colleagues (91) detected aromatase mRNA in the VTA of male and female rats, but only during early development. More recently, one study showed a behavioral effect of Cyp11a1 overexpression in the VTA, but not in the NAc (92). These results suggest that, if present in the VTA, Cyp11a1 affects reward-seeking behavior, but they did not demonstrate the importance of endogenous Cyp11a1 in the VTA. In the NAc, 3α-HSD and 5α-reductase type I, both involved in synthesizing DHT, are present in GABAergic medium spiny neurons of male mice (93). The steroidogenic acute regulatory protein (StAR), which is essential for *de novo* steroid synthesis, is also present in the NAc of mice (84).

**Figure 5:**
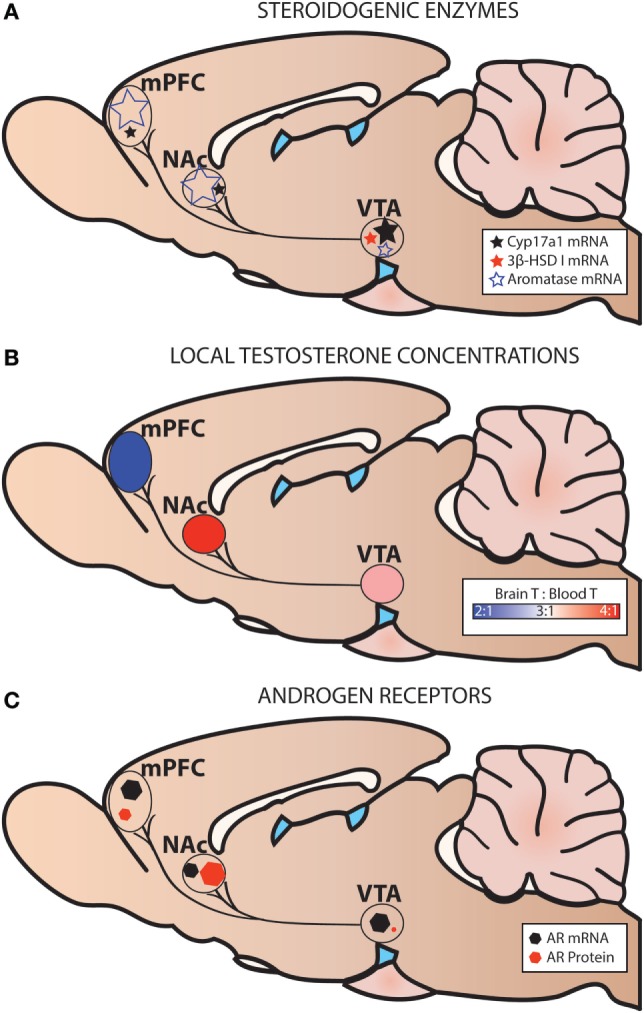
Steroidogenic enzymes, local testosterone concentrations, and androgen receptors (AR) in the mesocorticolimbic system of adult male rats. **(A)** Levels of steroidogenic enzyme mRNA are based on probe-based quantitative polymerase chain reaction (qPCR) assays (36). **(B)** Local testosterone (T) concentrations are based on Brain T: Blood T ratios in intact adult male rats [fed *ad libitum* or calorie restricted (36)]. **(C)** Levels of AR mRNA are based on probe-based qPCR assays (36), and levels of AR protein are based on immunohistochemistry (33). Levels of AR mRNA and AR protein are not shown relative to one another.

We have recently shown expression of *Cyp17a1, Cyp19a1* (aromatase), and *Hsd3b1* (3β-HSD type I) mRNA in microdissected mesocorticolimbic nodes in the adult male rat using exon-spanning, probe-based qPCR assays that are specific and sensitive [(36); Figure 5]. The VTA, NAc, and mPFC contained low levels of *Cyp17a1* mRNA, compared to the preoptic area/hypothalamus (POA/HYP). In the VTA, GDX decreased *Cyp17a1* mRNA at the 2 weeks time point. Compared to the VTA, the NAc and the mPFC contained much higher levels of aromatase mRNA. While GDX decreased aromatase mRNA in the POA/HYP, GDX had no effect on aromatase in VTA, NAc, or mPFC. 3β-HSD type I was expressed in trace amounts in the VTA, but was nondetectable in the NAc and mPFC. This is further evidence that the mesocorticolimbic system can synthesize androgens *de novo* from cholesterol or from circulating steroids (DHEA, progesterone).

**Figure 6:**
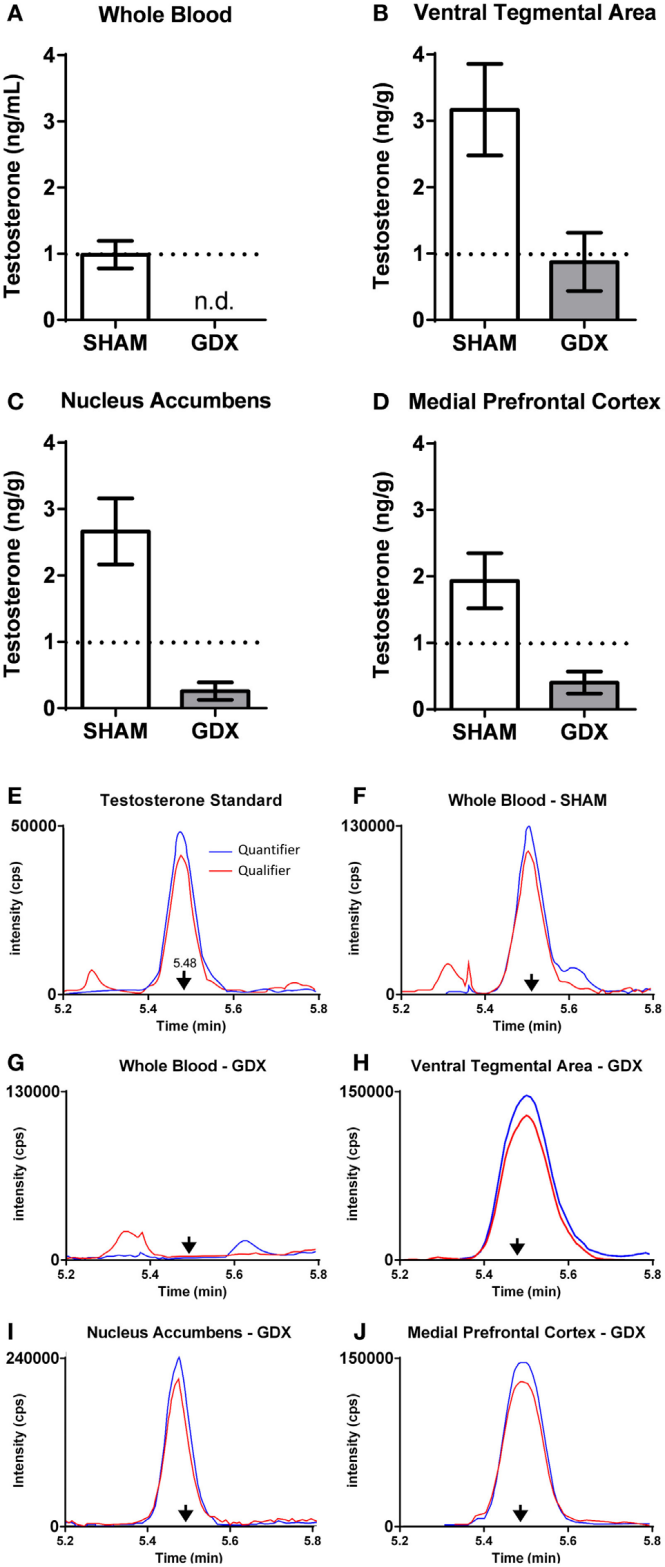
Testosterone is present in microdissected nodes of the mesocorticolimbic system of adult male rats at 6 weeks after GDX using LC–MS/MS. **(A–D)** Testosterone concentrations at 6 weeks after either SHAM surgery (*n* = 18–20) or GDX (*n* = 18–20) in the **(A)** whole blood, **(B)** ventral tegmental area (VTA), **(C)** nucleus accumbens (NAc), and **(D)** medial prefrontal cortex (mPFC). Values presented as mean ± SEM. **(E–J)** Representative chromatograms of testosterone quantifier ion (blue) and qualifier ion (red) for **(E)** testosterone standard (2 pg), **(F)** whole blood in a SHAM subject, **(G)** whole blood in a GDX subject, **(H)** VTA in a GDX subject, **(I)** NAc in a GDX subject, and **(J)** mPFC in a GDX subject. Arrows denote the retention time for testosterone. Note the differences in the intensity (counts per second, cps) on the *y*-axes. In **(G–J)**, samples are from different subjects, as not all GDX subjects had detectable testosterone in all brain regions. Adapted from Ref. (36). Abbreviations: GDX, gonadectomy; SHAM, sham surgery; n.d., nondetectable.

Using the contralateral side of the brain of the same subjects described above, we also examined steroid concentrations in mesocorticolimbic nodes *via* specific and ultra-sensitive liquid chromatography tandem mass spectrometry. Several results suggested local T synthesis. First, in sham-operated animals, T levels were 2–4× higher in the VTA, NAc and mPFC than in the blood (Figure 5B). Second, in all GDX subjects, T was nondetectable in the blood at 2 and 6 wks postoperatively (Figure 6). In the VTA, NAc and mPFC, T levels were lowered by GDX but nonetheless still detectable in ~50% of GDX subjects at 2 and 6 wks postoperatively (Figures 6A–D). Third, in subjects with detectable T, VTA T levels were similar in sham-operated and GDX subjects. Fourth, in GDX subjects, local T levels in the VTA might be driven by 3β-HSD type I, as *Hsd3b1* mRNA was positively correlated with T levels (*r* = 0.316). We did not detect other significant correlations between local T concentrations and steroidogenic enzymes in GDX animals, but we did not examine all androgenic enzymes (e.g., 17β-HSD, 3β-HSD type 2). Overall, these data suggest that androgen synthesis occurs in mesocorticolimbic nodes and partially compensates for the loss of circulating T in GDX animals. Moreover, the fact that T remains at physiologically relevant levels long after GDX suggests that it exerts a significant physiological effect. Future studies should examine how other androgenic enzyme isoforms may contribute to regulation of local androgen synthesis, as well as the physiological relevance of neurally produced androgens.

The presence of steroidogenic enzyme mRNA or protein does not necessarily indicate steroidogenic enzyme activity. Few studies have demonstrated steroidogenic enzyme activity in brain cells *in vitro* or *in vivo*. In male rats, T in the cerebral cortex and tegmentum (including the VTA) is metabolized into 5α-androstanolone *in vitro* (94). Zwain and Yen (95) established that neonatal rat astrocytes and neurons synthesize pregnenolone, DHEA, androstenedione, T, and E_2_ from precursors *in vitro*. Furthermore, steroidogenesis was reduced when the steroidogenic enzymes were pharmacologically inhibited or when transcription was inhibited. In humans, adult and fetal brains are capable of metabolizing T and androstenedione *in vitro* to E_2_ and T, respectively (96, 97). More recently, studies demonstrate that androgens and estrogens are synthesized *de novo* in male and female rat hippocampal slices (70, 71, 98). Steroidogenic enzyme activity, the mesocorticolimbic system, however, has not yet been examined. What is more, whether these neurally-produced steroids modulate behavior remains largely unexplored.

## AAS affect behavior *via* Action on the Mesocorticolimbic System

Recent studies have explored the consequences of androgen supplementation at supraphysiological (pharmacological) doses. This is relevant to the problem of AAS abuse. Importantly, when administered at pharmacological doses, AAS may act *via* different mechanisms from those under physiological conditions. AAS are performance-enhancing substances derived from T (99). The media focuses on AAS use among elite athletes and on steroid detection to ensure “fairness” in sport. In reality, use of AAS is far more widespread, and potential risks are only now becoming evident (100). As many as 3 million Americans have used AAS, which includes use in high schools, fitness centers, and “rejuvenation” clinics. A typical AAS user is a young man in his late teens or early 20s (100). Among U.S. high school students, 4–6% of boys have used AAS vs 1–2% of girls (101). This is comparable to the rates of crack cocaine or heroin use (101). It is estimated that AAS use among men in their 20s is even higher (100).

Commonly abused AAS include both aromatizable and non-aromatizable androgens (102). Elite athletes choose T because it is challenging to differentiate exogenous from endogenous sources (103). Rank-and-file users choose T because of its low cost and easy availability. Furthermore, most AAS users do not limit themselves to a single dose or type of steroid (104). Instead, users combine different steroids (“stacking”) in cycles of increasing and decreasing concentrations (“pyramiding”). AAS users take steroids orally, transdermally, or by intramuscular injection (105).

Recent research highlights a range of adverse health effects from chronic AAS abuse, including cardiovascular, hepatic, reproductive, and psychiatric dysfunction (105). However, the dangers of AAS abuse are not limited to the medical consequences of high-dose steroids themselves, but also result from risk-taking in non-social [e.g., drinking and driving (106)] and social contexts [e.g., aggression, sexual violence (107–110), and risky sex (106, 111, 112)]. Understanding the interplay of AAS and social behavior in risk-taking is particularly important in adolescents and young adults. This age group is strongly influenced by peer interactions (113, 114), exquisitely sensitive to rising levels of endogenous gonadal steroids (115), less risk-averse (116), and especially vulnerable to substance abuse (117). In part, this stems from adolescent immaturity in mPFC development (118).

Because it is not ethical to administer supraphysiological doses of AAS to normal volunteers, most of our knowledge of the behavioral effects of these drugs comes from studies of illicit users in the field and from animal studies. Furthermore, animal studies can explore consequences of AAS in an experimental context, where appearance and athletic performance are irrelevant. These studies have revealed that AAS appear to be rewarding and have potential to cause dependence. Rodents will voluntarily self-administer AAS orally (119) and by i.v. or i.c.v. injection (120). Moreover, they demonstrate tolerance, withdrawal, and fatal overdose with self-administration (121). T self-administration (i.c.v.) is blocked by the AR antagonist flutamide (121), although it appears that classical AR are not required for androgen reinforcement (122). The behavioral and physiological effects of supraphysiological doses of T resemble those of opioid overdose and are rapidly reversed by opioid antagonists (121). Likewise, many human AAS users meet DSM criteria for psychoactive substance dependence, including continued use despite negative side-effects, and withdrawal symptoms when steroids are discontinued (123).

The effects of AAS on reward and reinforcement strongly implicate involvement of the mesocorticolimbic system, since drugs of abuse act, in part, *via* DA release in NAc (124). Male rats form conditioned place preference (CPP) in response to intra-NAc infusion of T (125) or its metabolites (126), similar to the effects of DA-releasing drugs (127). Conversely, systemic or intra-NAc treatments with D_1_R and D_2_R antagonists block T-induced CPP (128, 129). Nonetheless, the manner in which androgens modulate DA release and signaling is still unclear. For example, acute administration of T does not induce NAc DA release (130), and in fact, AAS can reduce cocaine- or amphetamine-evoked DA release in NAc (131, 132). This latter finding is consistent with the observation that the acquisition of T self-administration is slow compared with cocaine or other addictive drugs (119). On the other hand, T upregulates the Fos protein, a marker of cellular activity, in regions of the mesolimbic DA system (133). Thus, the reinforcing effects of exogenous T may be due to its ability to modulate neural activity and DA signaling within the mesocorticolimbic circuit, but may do so without directly affecting DA release. Indeed, chronic AAS administration alters GABA_A_R subunit expression throughout the brain (including mesocorticolimbic regions), thus altering the physiological response to DA-independent GABAergic signaling (134, 135).

## Androgens Modulate the Neurochemistry and Structure of the Mesocorticolimbic System

Many androgen-dependent behaviors are mediated by neurochemical changes and neuronal activity in the mesocorticolimbic system. In several mammalian species, GDX of adult males diminishes expression of copulatory behavior, which can be restored by chronic T treatment (136). Copulatory behavior, particularly ejaculation, is correlated with a T-dependent increase in DA release in the NAc (137, 138). In this section, we will discuss how androgen deprivation, AAS, and androgen synthesis influence the neurochemistry and structure of the mesocorticolimbic system.

Most studies examining T regulation of the mesocorticolimbic system have focused on DAergic transmission in the NAc and mPFC. GDX alters DA tone in the mPFC of male and female rats (139). In the mPFC, GDX decreases basal DA after 4 days but increases it after 28 days. This is likely a result of GDX increasing bursting of VTA DA neurons, altering activity of mPFC efferents to the VTA, and gradually increasing TH in the VTA (140, 141). In contrast, in the NAc, basal DA is unchanged after GDX, but the DA metabolites 3,4-dihydroxyphenylacetic acid and homovanillic acid are increased after GDX (142). This finding suggests that GDX increases DA turnover in the NAc, which might indicate faster clearance of DA from the synapse and higher rates of DA signaling at baseline. GDX also modulates evoked electrophysiological and DAergic responses in the mPFC and NAc. In superfused striatal tissue, K^+^-simulated DA release was higher in GDX compared to GDX + T adult male mice (143). In the same study, reserpine, a drug that depletes DA, had the opposite effect, whereby DA release was higher in GDX + T male mice. This is in line with studies demonstrating that GDX affects storage, uptake, and/or synthesis of catecholamines in mesocorticolimbic nodes (142, 144) and helps to maintain NAc DA levels when exposed to methamphetamine (145).

Androgen-mediated structural plasticity and alterations in neurotransmitter receptor densities in the mesocorticolimbic system are other potential mechanism through which these hormones may alter cognitive/behavioral functions of this system. GDX decreases and high doses of T increase dendritic spine density in limbic regions, including the amygdala and HPC, in male rats (146, 147) and male monkeys (148). In a recent study, male rats were treated chronically with high-dose T, and brains were stained by Golgi–Cox to analyze neuronal morphology in medium spiny neurons of nucleus accumbens shell (NAcS) (149). T decreased spine density throughout the dendritic tree in the NAcS. However, T treatment did not affect total spine number, dendritic length, or arborization. Similarly, in the mPFC, GDX reduces and DHT increases dendritic spine formation in male mice (150). The effect of DHT on dendritic spine formation was reduced, but not absent, in GDX testicular feminization mutant male rats (a naturally occurring mutant with severely attenuated AR binding capacity), which suggests both androgenic and non-androgenic influence on synaptic remodeling.

Androgens can influence the function of the mesocorticolimbic nodes by their local metabolism to more potent androgens (e.g., T→DHT), further metabolism to weak androgens (e.g., T→DHT→3α-androstanediol), or metabolism to estrogens (T→E_2_). 3α-androstanediol, for example, has weak androgenic effects but also acts as a robust and rapid neuromodulator *via* allosteric binding to GABA_A_R (66, 151). Indeed, 3α-androstanediol in the NAc facilitates CPP (a DA-dependent behavior) in rodents, likely through allosteric agonism of GABA_A_R in GABAergic medium spiny neurons (152). Concurrently, the aromatization of T into E_2_ may also influence activity in the mesocorticolimbic system. E_2_ decreases striatal DA transporter density, enhances DA synthesis and degradation (153, 154), and downregulates DA binding to D_2_R in the NAc (155). In contrast, systemic treatment with the aromatase inhibitor letrozole decreases basal DA turnover in the mPFC of male and female adult rats (156). The regulation of DA turnover in the brain may be directly related to changes in phasic DA signaling. Indeed, direct pulsatile application of E_2_ to striatal slices induces DA release (157) and enhances K^+^-mediated DA release (158). Patch clamp analysis of ion transfer across the membrane in dissociated NAc medium spiny neurons demonstrated that there is a prompt diminution of Ca^++^ currents in response to acute E_2_ (159). Taken together, these data suggest that local production of E_2_ in males and females modulates DA signaling and postsynaptic neural excitability in the mesocorticolimbic system.

Few studies have examined whether neurosteroids produced in the mesocorticolimbic system influence neurochemistry. Pharmacological inhibition of Cyp17a1 regulates a DA-dependent behavior [prepulse inhibition (PPI)], but the study did not examine the direct effect on DA signaling (160). Inhibition of Cyp17a1 would decrease both androgen and estrogen signaling. DHEA, a product of Cyp17a1, is present in human, but not in laboratory rat or mouse, circulation. DHEA has a wide range of neurochemical effects, but the source of DHEA is rarely determined (161). The mesocorticolimbic system is sensitive to DHEA. For example, DHEA decreases the activity of monoamine oxidase, an enzyme necessary for the degradation of monoamines, in the NAc in male rats *in vivo* and *in vitro* (162). Pharmacological inhibition of 5αR suggests that DHT influences neurochemistry, particularly DA signaling, in the mesocorticolimbic system (163–165). Overall, these data suggest neurosteroids regulate DA turnover and DA signaling in the mesocorticolimbic system, which is important for regulating executive functions.

## Androgens Regulate Executive Function

Clinical and preclinical evidence suggest that hypogonadism and GDX have deleterious effects on executive functioning, which can often be ameliorated with androgen replacement. Furthermore, excessive androgen exposure (e.g., AAS) during adolescence and/or adulthood has detrimental effects on executive functioning. We also examine evidence that the brain compensates for a decrease in circulating androgens by increasing local androgen synthesis in the mesocorticolimbic system to mitigate deficits in executive functioning. These studies support the hypothesis that there is an optimal level of androgen signaling within the mesocorticolimbic system for proper executive functioning.

### Low Androgen Signaling

Andrew and Rogers (166) were among the first to demonstrate that androgens affect executive function. In a foraging paradigm, young male chicks treated with T pecked grains of a familiar color and ignored unfamiliar, novel-colored grains, while vehicle-treated chicks demonstrated behavioral flexibility and pecked both grain colors without bias (166). The authors used the term “persistence” (also called “perseveration”) to describe the inability to stop using a response strategy when it is no longer relevant or advantageous. Rogers (167) then showed that antiandrogen treatment or GDX decreased persistence in adult male chickens, whereas systemic T replacement in GDX chickens reinstated persistence. Subsequent studies in adult male rodents revealed that GDX or an antiandrogen reduced persistence, supporting the initial findings in birds (168, 169). GDX also decreases male persistence during social investigation of female conspecifics, suggesting that T increases male persistence in gaining access to potential mates (170).

T increases perseveration in operant conditioning tasks that require behavioral flexibility. Using a reversal learning task, van Hest and colleagues (171) demonstrated that GDX male rats perseverated less on the previously reinforced lever, while administration of T to GDX subjects displayed the highest rates of perseveration. Additionally, GDX male rats exposed to a conditional discrimination task in a T-maze made fewer errors during the reversal phase (i.e., decrease in perseverative errors) compared to intact subjects (172). On a delayed spatial alternation test, GDX subjects made less perseverative errors than intact subjects, but only after a delay of 6 sec or more, suggesting a concurrent deficit in working memory (173).

In men, declines in executive functioning and visuospatial ability are the most commonly reported adverse cognitive effects of androgen deprivation therapy [ADT (174)]. ADTs are administered to nearly 50% of prostate cancer patients (175) and include GnRH analogs (e.g., Histrelin), AR antagonists (e.g., flutamide), and androgenic enzyme inhibitors (e.g., abiraterone, a Cyp17a1 inhibitor) [see (176) for review]. Many ADTs decrease systemic T but might not decrease local androgen synthesis equally across tissue types (e.g., GnRH analogs). If a clinical study includes only subjects on ADTs that inhibit androgen synthesis and/or signaling in the brain, which is often not the case, then the effects of ADTs on executive function might be more clear (see below).

There is contradictory evidence on the effect of ADTs on executive functioning. For example, ADT is associated with deficits in attention and cognitive control [i.e., Trail Making B task, Stroop Interference Test (177)]. Furthermore, ADT is associated with decreases in gray matter volume in the dorsolateral and frontopolar PFC (178) and decreased neural activity and connectivity in the mPFC during tasks requiring inhibitory control (179). ADT is also associated with changes in impulsivity, emotional lability, and working memory, compared to matched non-ADT subjects (180). These findings suggest that ADT disrupts PFC function, an area particularly sensitive to androgens in males (33, 39).

However, other studies have not found an association between ADT and executive function (181–183). A recent meta-analysis of the effects of ADT on a variety of cognitive functions found that only visuospatial ability was reliably affected by ADT, whereas assays of executive functioning (e.g., Trail Making Test B, Stroop Interference Task) did not detect any significant differences (184). These findings are in line with a study in menopausal women with low T who were then treated with T and did not show changes in a range of executive functions compared to untreated subjects (185). However, the assessment tools for these studies might not be sensitive enough to detect small, yet important, changes in executive functions [e.g., (186)]. In addition, studies on ADT and executive function may lack statistical power, neglect confounding variables (183, 187), or include subjects who have not received ADT for enough time [ADT is usually administered for 2–3 years (181)]. These issues reduce the ability to detect effects of ADT on executive functioning. For example, Alibhai and colleagues (181) conducted a small study that regularly administered a battery of neuropsychological tests to ADT patients for 36 months, and ADT was not associated with deficits in cognitive flexibility or working memory. However, this study might not have utilized cognitive tests sensitive enough to detect PFC-specific deficits in executive functioning [e.g., Iowa Gambling Task (IGT)]. Moreover, 95% of ADT subjects in this study were using GnRH analogs as their sole ADT, which may not affect androgen synthesis in the brain, as would androgenic enzyme inhibitors.

### High Androgen Signaling

**Figure 7:**
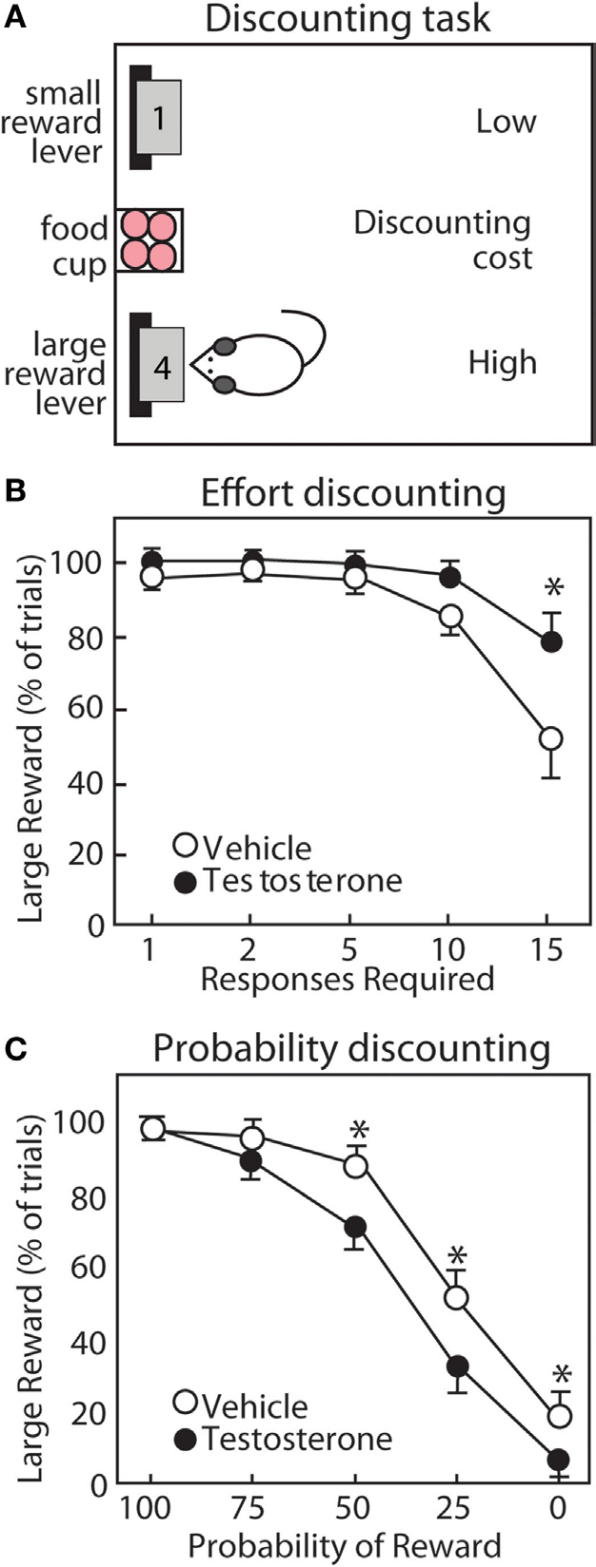
Testosterone influences discounting behavior of male rats. **(A)** Operant task for discounting behavior. Rats choose between two levers. The small reward lever delivers 1 pellet with minimal cost. The large reward lever delivers four pellets with increasing cost throughout the session. **(B)** For effort discounting behavior, the response requirement (number of lever presses) increases. **(C)** For probability discounting, the large reward is delivered with decreasing probability. Compared with vehicle controls (open circles), testosterone (closed circles) increases preference for the large reward lever in effort discounting, but reduces preference for the large reward lever in probability discounting. Adapted with permission from Ref. (190). Values presented as mean ± SEM. *p ≤ 0.05.

**Figure 8:**
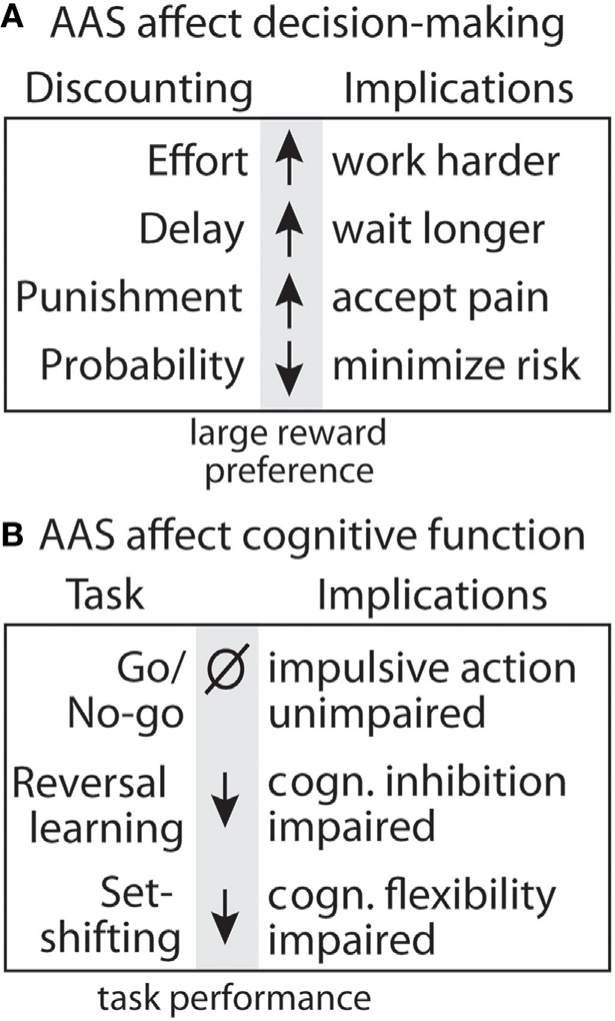
Summary of effects of anabolic-androgenic steroids (AAS) on decision making and cognitive function. **(A)** Testosterone effects on discounting behavior for effort (190), delay (191), punishment (192), and uncertainty (190). **(B)** Testosterone effects on cognitive function and motor impulsivity in the go/no-go task (192), cognitive inhibition in the reversal learning task, and cognitive flexibility in the set-shifting task (188).

Supraphysiological levels of androgens typically seen with AAS use also impair executive function. This has been explored in rats treated with supraphysiological levels of T and trained to work for food reward (sugar pellets) in an operant chamber (Figures 7 and 8). T-treated rats display deficits in different forms of cognitive flexibility, including reversal learning and extra-dimensional set-shifting (188). These rats take longer to shift their behavior when stimuli associated with rewards are reversed, or when rats must employ a novel discrimination strategy to obtain rewards. Importantly, set-shifting behavior is dependent upon D_1_R in NAc (12), and AAS reduce NAc D_1_R (189).

Anabolic-androgenic steroids also alter different forms of cost/benefit decision making in operant discounting tasks. In these tasks, rats choose between two retractable levers, one of which is associated with a smaller, easily obtainable reward (1 pellet) and the other with a larger reward (3 or 4 pellets) associated some cost. These costs can include effort, delay, punishment, and probability, which results in discounting of the larger reward (i.e., making it less desirable; Figure 8). AAS and DA have site- and task-specific effects on discounting behavior. In particular, AAS do not cause impulsivity with a consistent preference for small rewards, nor do they produce a “win-at-all-costs” strategy that always favors the large reward. Instead, there is a selective effect of AAS. Specifically, AAS-treated rats are less sensitive to effort (188), punishment (192), and delay (191), but are more sensitive to uncertainty (190). In particular, AAS may diminish sensitivity to future negative consequences, even as they render users more sensitive to unpredictable outcomes.

A wealth of studies has mapped the neurotransmitters and brain regions responsible for discounting behavior using systemic treatment with neurotransmitter receptor agonists and antagonists and selective inactivation of NAc subregions. As discussed previously, D_1_R and D_2_R each promote preference for the large reward in effort discounting (delivery of the large reward requires more lever presses) and probability discounting [delivery of the large reward is uncertain (193)]. Studies using inactivation of NAc subregions have revealed that effort discounting is regulated by the NAc core (NAcC), while probability discounting is regulated more prominently by the NAc shell [NAcS (194, 195)]. These findings align with modulation of D_1_R and D_2_R in NAc subregions by the AAS nandrolone (189) and with recent studies of effort and probability discounting in response to high-dose T (190). T reduces preference for larger reward during probability discounting (190), and AAS reduce DA receptors in NAcS (189). Conversely, T treatment increases preference for the large reward during effort discounting (190), and nandrolone increases D_2_R in NAcC (189). Thus, AAS might reduce sensitivity to effort during effort discounting by increasing D_2_R in NAcC.

In animal studies, it is interesting that AAS selectively alter elements of risk-taking and impulsivity. In probability discounting, risk is reflected by the potential for reward omission, and T makes rats more risk-averse, an effect that may be driven by a reduction in NAc D_1_R. At the same time, they are less risk-averse in punishment discounting, whereby they risk a footshock with delivery of the large reward (190). Together, these results reveal a nuanced effect of supplemental T to increase sensitivity to reward omission and simultaneously decrease responsiveness to punishment. A similar picture emerges in assessment of how T alters different aspects of impulsivity. T has no effect on impulsive actions as measured in a go/no-go task (192), wherein rats must switch between initiating and inhibiting a response to obtain rewards. However, the same study showed that T reduces impulsive choice, assessed with a delay discounting task, in that it increases the subjects’ willingness to wait for a large, delayed reward. Given the complex manner in which increasing androgen activity can influence various forms of decision making, it is unlikely that the effects of these treatments are driven by uniform increases or decreases in mesolimbic DA activity. Rather, these findings suggest that the manner in which T influences the behavioral functions depends in part on the specific costs that are being evaluated and the underlying corticostriatal circuitry that is recruited in guiding these decisions.

Studies of executive function in human AAS users are limited and restricted to male subjects. AAS abusers show impaired visuospatial working memory compared to non-users, similar to deficits seen in ADT (184), and the level of impairment is correlated with lifetime AAS use (196). A variety of evidence further implicates androgens and AAS in risk-taking behavior in humans. In a study of American high school students, AAS use was associated with risky sex, drinking and driving, carrying a weapon, and not wearing a helmet or seat belt (106). Psychological evaluations of human users have also implicated AAS in impaired decision making, which may stem from feelings of invincibility (197). Deaths among AAS users show high rates of homicide, suicide, and drug overdose (110). These possible effects of AAS abuse on risk-taking in humans might be similar to punishment discounting in rats, in that androgens increase the appetite for reward despite a risk of punishment.

Risk taking induced by AAS has a potentially dangerous social dimension as well. AAS use has been implicated in several violent murders (107–110). In surveys of current AAS users and in studies of human volunteers, increased aggression is the most consistent behavioral effect of high-dose AAS exposure in humans (103, 105). Compared with non-users, AAS users report increased sex drive (198) and increases in risky sexual behaviors [i.e., increased numbers of partners, infrequent condom usage (111)], as well as unprotected anal intercourse among HIV-positive gay men (112). Among American high school students, AAS use correlated with not using a condom and a history of sexually transmitted disease (106). Thus, a key danger of AAS abuse is the likelihood that users will engage in behaviors that harm themselves and those around them.

### Individual Variation in Circulating Androgen Levels and Executive Functioning

In normal, healthy individuals, circulating levels of androgens vary dramatically, allowing for correlational analyses of androgen levels and executive function. Interestingly, perseveration and risky behavior are positively correlated with endogenous androgens in adolescence and adulthood, similar to findings from animal studies (199). For example, adolescent males that exhibit external signs of high T (e.g., hirsutism) perform better on simple repetitive tasks than those without such external signs, independent of cognitive ability (200). This early study suggested a positive correlation between endogenous androgens and persistence. Furthermore, higher androgen levels in pubertal boys correlate with a greater probability of lifetime ethanol use (201). Therefore, circulating androgens may enhance ethanol effects on behavior, potentially increasing risk-taking in one or both sexes. To address this possibility, a study compared GDX male and female rats with and without hormone replacement in the probability discounting task, to investigate the influence of ethanol and gonadal steroids on the response to uncertainty (202). At baseline, GDX + T males showed a greater preference for the large reward than GDX males. Ethanol further increased large reward preference, but only in males. These results suggest that both ethanol and T at normal physiological levels increase tolerance for a large uncertain reward.

In adults, men with higher T are more likely to choose cards from decks offering large monetary gains paired with larger, infrequent losses in the IGT, a probabilistic, risk-based decision making task (203). This result is similar to patients with damage to the OFC and ventromedial PFC (204, 205). As a result, men with higher T earned less money throughout the session, relative to men with lower T. High levels of endogenous T also correlate with economic risk-taking outside of the lab. In a study of London stock traders, morning T levels predicted risk-taking throughout the day (206). In young and menopausal women, T is not associated with changes in any measure of executive function (207–209). Taken together, such studies suggest that individual variation in systemic T levels in males, but not females, is correlated with specific aspects of executive function.

### Neuroandrogens and Executive Function

In addition to acting as endocrine signals, androgens also act as intracrine, paracrine, and autocrine signals. Specific nodes within the mesocorticolimbic system might require a particular androgen concentration to function appropriately. Similarly, McEwen and Wingfield (210) posited that local glucocorticoid signaling is tightly regulated to alleviate allostatic load imposed by high circulating glucocorticoid levels. Studies utilizing extreme changes to circulating androgen concentrations, such as AAS, GDX, or ADT demonstrate the importance of systemic androgens for executive function, but they can not reveal the role of local androgen synthesis. As discussed above, local levels of T in the mesocorticolimbic system vary greatly from circulating levels and from one neural node to another. Levels of T are often two or more times higher in the mesocorticolimbic system than in the blood in intact animals, and T is still present in the mesocorticolimbic system at 6 weeks after GDX (36). These results suggest that local T synthesis is important for neural activity in the mesocorticolimbic system and executive functioning.

There are few data on how the local production of androgens in the mesocorticolimbic system influences executive functioning. Several studies report changes in T precursors or androgenic enzymes in the mesocorticolimbic system of patients with mood disorders or in animal models of depression (211–213). Depression is frequently marked by deficits in executive functions (214, 215). In fact, clinicians refer to a disorder that occurs in geriatric populations as “depression-executive dysfunction syndrome” (214, 216). Low circulating DHEA and DHEA-S levels are correlated with depression in aged human populations, and DHEA has been suggested as treatment for depression (217, 218). In a rodent model of childhood depression, DHEA levels are lower in the VTA and NAc (but not amygdala or hypothalamus), suggesting mesolimbic-specific regulation of androgens (211). Expression of several steroidogenic enzymes are altered in post-mortem analyses of depressed individuals, which include a decrease of 5α-reductase type I in the PFC (213) and Cyp17a1 in the anterior cingulate cortex [ACC (212)], and an increase in hydroxysteroid sulfotransferase 2A1 (HST, Figure 1) in the ACC and StAR in the dorsolateral PFC (212). Changes in the expression of these specific steroidogenic enzymes suggest active androgen synthesis and metabolism in the VTA, NAc, and PFC.

Systemic administration of steroidogenic enzyme inhibitors that cross the blood–brain barrier hint at the role of neuroandrogens in modulating executive function. Using set-shifting and reversal learning tasks, the Soma laboratory has recently found that chronic systemic administration of abiraterone (a Cyp17a1 inhibitor) enhances behavioral flexibility in intact and gonadectomized subjects [unpublished results (219)]. Furthermore, systemic administration of letrozole, an aromatase inhibitor, increases risk-taking behavior in human males (220) and improves working memory in female rats (221). In particular, the study by Goudriaan et al. (220) administered letrozole to healthy men for 1 week and tested executive function and risk-taking before and after treatment. Importantly, this treatment was used to increase circulating T, but potentially influenced steroidogenesis in the mesocorticolimbic system. Letrozole-treated subjects demonstrated an increase in risk-taking on the Balloon Analog Risk-Taking task, but not the IGT or Game of Dice, compared to their baseline and estrogen-treated subjects. These findings highlight the importance of using a variety of sensitive neurocognitive assays to detect changes in executive function. This study, along with the studies of behavioral flexibility in male rats and working memory in female rats, suggests that androgens and local androgen synthesis, and not E_2_ or local androgen metabolism, have the most profound effects on executive functioning.

There have been no studies, to our knowledge, that directly (e.g., i.c.v. steroidogenic enzyme inhibitor) manipulated neural androgen synthesis and examined executive functioning. The most relevant studies examined the effects of androgenic enzyme (i.e., Cyp17a1 and 5αR) inhibitors on PPI of the acoustic startle reflex and DA signaling in the NAc (160, 163, 165). Frau and colleagues (160) administered apomorphine (a non-selective DA agonist; i.p.) to male rats to cause a deficit in PPI. The effects of apomorphine on PPI were attenuated by microinjecting (i.c.v.) the Cyp17a1 inhibitor abiraterone. Along with studies using systemic finasteride [5αR inhibitor (163, 164, 222, 223)], these results suggest that local androgen synthesis regulates DA signaling in the mesocorticolimbic system and DA-dependent behaviors. While these studies are informative, there still remains an important gap in our understanding of how neural androgen production specifically influences executive functioning.

## Conclusion

Androgens influence a variety of behaviors and cognitive functions, which include executive functioning. Converging lines of evidence suggest that androgens can influence executive functioning *via* actions on the mesocorticolimbic system. Multiple nodes of the mesocorticolimbic system (VTA, NAc, mPFC, and OFC) contain AR and ER. Emerging evidence suggests that multiple nodes of the mesocorticolimbic system also locally synthesize androgens, estrogens, and other steroids. However, the physiological role of these neuroandrogens still remains to be determined. Reducing endogenous androgens (GDX, ADT) and administering exogenous androgens (AAS) alter the neurochemistry (e.g., DA signaling) and cytoarchitecture of the mesocorticolimbic system. In animal studies, both a reduction of endogenous androgens and pharmacological administration of exogenous androgens lead to alterations in behavioral flexibility and inhibitory control. In human studies, evidence suggests that ADT or AAS abuse can also lead to deficits in executive functioning. Future studies should investigate the roles of systemic and locally produced androgens in the mesocorticolimbic system and cognition. Taken together, such studies broaden our understanding of androgen regulation of behavior to include decision making and executive function, and also highlight neurosteroid and AAS action within the mesocorticolimbic system.

## Author Contributions

All authors listed have made a substantial, direct and intellectual contribution to the work and approved it for publication.

## Conflict of Interest Statement

The authors declare that the research was conducted in the absence of any commercial or financial relationships that could be construed as a potential conflict of interest.

## Abbreviations

17β-HSD, 17β-hydroxysteroid dehydrogenase; 3β-HSD, 3β-hydroxysteroid dehydrogenase/isomerase; 5αR, 5α-reductase; AAS, anabolic androgenic steroids; ACC, anterior cingulate cortex; ADT, androgen deprivation therapy; AR, androgen receptor; CPP, conditioned place preference; D_1_R, dopamine receptor D_1_; D_2_R, dopamine receptor D_2_; DA, dopamine; DAT, dopamine transporter; DHEA, dehydroepiandrosterone; DHEA-S, dehydroepiandrosterone sulfate; DHT, 5α-dihydrotestosterone; E_2_, 17β-estradiol; ER, estrogen receptor; ERα, estrogen receptor α; ERβ, estrogen receptor β; GABA, γ-aminobutyric acid; GABA_A_R, Type A γ-aminobutyric acid receptor; GDX, gonadectomy; GPER1, G protein–coupled estrogen receptor 1; GPRC6A, G protein–coupled receptor family C group 6 member A; HST, hydroxysteroid sulfotransferase 2A1; i.c.v., intracerebroventricular; i.v., intravenous; IGT, Iowa Gambling Task; ir, immunoreactivity; mAR, membrane-associated androgen receptor; mER, membrane-associated estrogen receptor; mPFC, medial prefrontal cortex; mPFC-PL, prelimbic medial prefrontal cortex; NAc, nucleus accumbens; NAcC, nucleus accumbens core; NAcS, nucleus accumbens shell; NMDA-R, *N*-methyl-D-aspartate receptor; OFC, orbitofrontal cortex; PCR, polymerase chain reaction; PFC, prefrontal cortex; POA/HYP, preoptic area/hypothalamus; PPI, prepulse inhibition; qPCR, quantitative polymerase chain reaction; StAR, steroidogenic acute regulatory protein; T, testosterone; TH, tyrosine hydroxylase; TSA, tyramide signal amplification; VTA, ventral tegmental area; ZIP9, Zrt- and Irt-like protein 9.
